# Optimal Use of Titanium Dioxide Colourant to Enable Water Surfaces to Be Measured by Kinect Sensors

**DOI:** 10.3390/s20123507

**Published:** 2020-06-21

**Authors:** Andrew Nichols, Matteo Rubinato, Yun-Hang Cho, Jiayi Wu

**Affiliations:** 1 Department of Civil and Structural Engineering, The University of Sheffield, Sir Frederick Mappin Building, Mappin Street, Sheffield S1 3JD, UK; yun-hang.cho@sheffield.ac.uk (Y.-H.C.); jiayi.wu@sheffield.ac.uk (J.W.); 2 School of Energy, Construction and Environment, Faculty of Engineering, Environment and Computing, Coventry University, Coventry CV1 5FB, UK; matteo.rubinato@coventry.ac.uk; 3 Centre for Agroecology, Water and Resilience, Coventry University, Wolston Lane, Coventry CV8 3LG, UK

**Keywords:** kinect, water surface measurement, remote sensing, free surface, surface gravity wave

## Abstract

Recent studies have sought to use Microsoft Kinect sensors to measure water surface shape in steady flows or transient flow processes. They have typically employed a white colourant, usually titanium dioxide (TiO_2_), in order to make the surface opaque and visible to the infrared-based sensors. However, the ability of Kinect Version 1 (KV1) and Kinect Version 2 (KV2) sensors to measure the deformation of ostensibly smooth reflective surfaces has never been compared, with most previous studies using a V1 sensor with no justification. Furthermore, the TiO_2_ has so far been used liberally and indeterminately, with no consideration as to the type of TiO_2_ to use, the optimal proportion to use or the effect it may have on the very fluid properties being measured. This paper examines the use of anatase TiO_2_ with two generations of the Microsoft Kinect sensor. Assessing their performance for an ideal flat surface, it is shown that surface data obtained using the V2 sensor is substantially more reliable. Further, the minimum quantity of colourant to enable reliable surface recognition is discovered (0.01% by mass). A stability test shows that the colourant has a strong tendency to settle over time, meaning the fluid must remain well mixed, having serious implications for studies with low Reynolds number or transient processes such as dam breaks. Furthermore, the effect of TiO_2_ concentration on fluid properties is examined. It is shown that previous studies using concentrations in excess of 1% may have significantly affected the viscosity and surface tension, and thus the surface behaviour being measured. It is therefore recommended that future studies employ the V2 sensor with an anatase TiO_2_ concentration of 0.01%, and that the effects of TiO_2_ on the fluid properties are properly quantified before any TiO_2_-Kinect-derived dataset can be of practical use, for example, in validation of numerical models or in physical models of hydrodynamic processes.

## 1. Introduction

The dynamic pattern on the free surface of open channel flows varies according to the flow rate and boundary conditions. Previous research has found that turbulence generated near the bottom of a channel by the bursting phenomenon is transferred towards the water surface [1]. More recent work has clarified somewhat the link by showing that water surface fluctuations in shallow flows can be associated with the underlying velocity field and turbulence, which can in turn be related to the flow conditions, boundary conditions and hydraulic processes [2,3,4,5,6,7]. Despite this, there is still a lack of detailed explanation regarding the link between free-surface features and the underlying flow conditions [1,8,9,10]. Investigating the relationship between the underlying flow and the free surface is crucial as it has the potential to enable remote, nonintrusive measurement of flow processes. It could therefore find applications in remote flow monitoring, sediment entrainment studies and pollutant transport.

However, this potential can only be realised via an ability to accurately characterise the three-dimensional dynamic surface roughness patterns of turbulent flows. Several techniques exist for measuring water surface position [11,12,13,14,15,16,17,18], but these techniques are either prohibitively expensive and complex, limited in their spatial resolution or difficult to implement in the field, or generally measure only in one or two dimensions.

Based on limitations identified by previous studies of other techniques (e.g., point gauges, surface profiling systems [19,20,21,22] or laser scanners [23]), there is a need for measurement techniques that can be affordable, easy to use and accurate at the same time. To date, Microsoft Kinect sensors have been used for a range of scientific applications including: (i) 3D indoor mapping [24], (ii) real-time 3D modelling [25], (iii) health care [26,27], (iv) surveillance [28], (v) earth sciences [29], (vi) morphological measurements [30,31] and (vii) musculoskeletal disorders [32]. Gonzalez-Jorge et al. [33] demonstrated how the depth accuracy of these sensors is dependent on the distance to the measured objects and other studies have explored the differences in accuracy and reliability between the two sensors for common 3D reconstruction applications [34,35,36,37,38]. Gonzalez-Jorge et al. [34] highlighted how Kinect Version 2 (KV2) performs more accurately in indoor conditions, and Wasenmüller and Stricker’s comprehensive comparison of KV1 and KV2 [35] also found KV2 to provide superior data quality. However, no direct comparison between KV1 and KV2 has been presented for dynamic water surfaces, which are ostensibly smooth and reflective.

Nichols and Rubinato [39] first presented an initial examination of the use of Kinect sensors for low-cost 3D measurements of flowing water surfaces, and other studies have since applied the same technique [40,41,42,43,44]. While the popularity of using Kinect sensors for hydraulic experiments is growing, more research is needed to explore this potential and limitations of Kinect technology under multiple hydraulic and experimental conditions. Nichols and Rubinato [39] showed that the Kinect has the potential to measure gravity waves and may also be used to measure turbulence-generated free-surface roughness. They showed that titanium dioxide (TiO_2_) could be used to colour the water and cause its surface to become opaque so that the infrared signals from Kinect sensors can be reflected, and Martinez-Aranda et al. [40,41] subsequently employed the same methodology to measure free-surface shape during dam break flows and shallow turbulent flows. A very limited number of studies have used the Kinect–TiO_2_ methodology, and these are summarised in Table 1. Very few studies specify the concentration used, or the specific type of TiO_2_ used.

The use of colourant for water surface visualisation is not unprecedented for other optical measurement systems. Tsubaki and Fujita [12] proposed a new stereoscopic measurement for fluctuating free surfaces by mixing an unspecified amount of an unspecified white dye into the water to make the water opaque so that the surface appears solid. Cobelli [45] measured the free-surface deformation by projecting a fringe pattern by a video projector and recording it using a digital camera. A “standard, highly concentrated titanium dioxide pigment paste” was reportedly used at a 0.5% concentration and was said to “not affect water’s hydrodynamical properties” but without proof or justification. The concentration was said to be a compromise between diluteness and high fringe pattern contrast, and it was reported that below the saturation point of 10% by volume, phase separation (forming multiple layers with different properties) will not occur. Other colourants have also been explored. Aureli et al. [46] detected the topography of water surfaces based on light absorption by the water body. They coloured the water by adding methylene blue, which acts as a variable-density filter. The colouring agent concentrations were chosen to achieve the maximum sensitivity for all laboratory test conditions, but the actual concentrations used were not reported. They found that a reduction in the colour agent concentration can lead to reduction of the overall sensitivity. Chatellier et al. [47] mixed a mass concentration of 10% polyoxide ethylene powder. Cang et al. [48] measured the wave height by a binocular camera, using an unspecified colourant at an unspecified concentration to turn the water “milky white” to decrease the penetration of light.

Despite all the insights provided, there is still only limited evidence regarding the effect of TiO_2_ concentration on the fluid properties, such as surface tension and viscosity. This can intrinsically affect the very phenomena being studied. Tadeu [49] showed that rutile TiO_2_ increases the viscosity of water by over 10% at just a 1% concentration and decreases surface tension by almost 30% at a concentration of 1%. Przadka et al. [50] investigated the use of TiO_2_ in Fourier profilometry, another optical method of surface reconstruction. They found that the rutile TiO_2_ indeed did affect surface wave behaviour on water, while anatase TiO_2_ only marginally affected the wave behaviour. However, this was for a transient wave of several millimetres in amplitude; many applications require the examination of continuous waves with smaller amplitudes, and the effect of TiO_2_ in these conditions has never been studied. 

No study has ever sought to establish an optimal concentration of TiO_2_ for practical use with Kinect sensors, which use infrared rather than visible light, infrared being known to perform differently in optical sensing applications [51,52]. Nor has any paper explored the differences between the two common versions of the Kinect sensor in the application of water surface measurement. 

This paper aims to establish the optimal TiO_2_ concentration and sensor choice, and present evidence that the fluid properties can be substantially affected by the TiO_2_ and thus this effect demands deeper study before TiO_2_–Kinect data can be reliably interpreted. 

This paper is organised as follows: Section 2 presents a comparison of the two sensor types and establishes a minimum TiO_2_ concentration for reliable use. It also explores the post-processing techniques that can be used to improve data quality as a function of spatial and temporal accuracy. Section 3 explores the effect of TiO_2_ concentration on fluid properties. Finally, in Section 4, conclusions are presented and recommendations for future work are given.

## 2. Sensor Accuracy and Minimum Colourant Concentration

This section describes the experimental design, equipment and methodology applied to calibrate and validate the Microsoft Kinect Version 1 (KV1) and Microsoft Kinect Version 2 (KV2) measurements, and the comparative results of the sensors’ performance. 

### 2.1. Kinect Sensors

**Figure 1:**
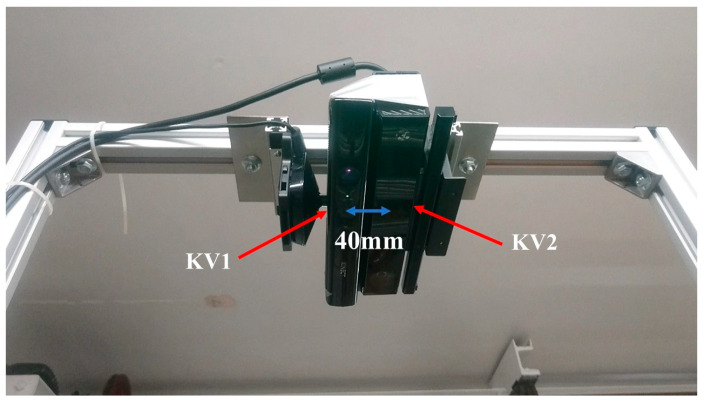
KV1 (**left**) vs. KV2 (**right**) used for this study.

KV1 contains an infrared (IR) imaging emitter coupled with an IR camera. The 3D images obtained are produced from a light coding technique. The IR emitter generates a speckle pattern on the object under study; this object needs to be opaque and ideally matte in order to diffuse the projected pattern which will be captured by the IR sensor. The image received by the sensor is compared with the original pattern by an on-board processor, which uses the relative distortion to associate depth information to each pixel [24,53]. In contrast, KV2 uses the Time-of-Flight technique (ToF) which is a method for measuring the distance between a sensor and an object based on the time difference (or phase difference) between the emission of a signal and its return to the sensor, after being reflected by the object. This type of technique is often used on LIDAR sensors for autonomous vehicles. The resolution of the depth map for KV1 is 480 × 640 and for KV2 is 424 × 512. Typically, depth and RGB data are recorded by both devices at 30 frames per second (fps), which has been shown to be reliable by previous studies [35,39,44]. The data presented here were also recorded at 30 fps. KV1 and KV2 are displayed in the experimental setup in Figure 1. 

### 2.2. Kinect Data Calibration

**Figure 2:**
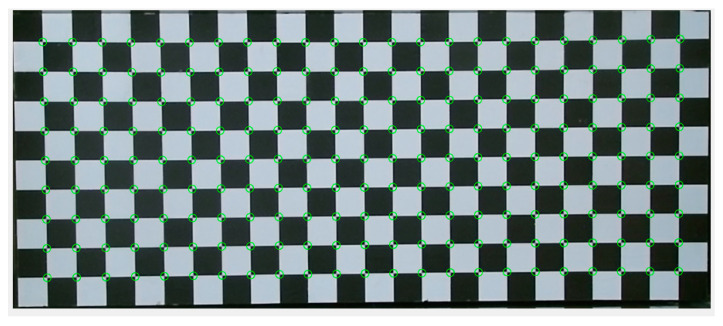
Chequerboard points identification for the spatial calibration; image shown is the raw image prior to dewarping.

A preliminary procedure was undertaken to calibrate KV1 and KV2. The devices were positioned facing downwards (vertical view direction, see Figure 1) with their front faces at a distance of 1.5 m above the floor of a rectangular wave tank with cross-section of 355 mm by 210 mm. This provided an ideal field of view with high resolution and high accuracy due to being within the optimal distance [34]. The sensors were installed close to each other as shown in Figure 1, with a horizontal separation of 40 mm between their infrared detectors. For calibration, the tank was temporarily removed and a 500 mm by 300 mm chequerboard pattern was placed at a range of vertical positions to horizontally and vertically calibrate the KV1 and KV2. A spatial calibration function known as “fitgeotrans” was used to calculate a geometric transformation from the detected calibration board vertices (see Figure 2) to an orthogonal grid whereby 1 pixel = 1 mm horizontally. This allowed the effects of lens distortion to be mitigated by dewarping the images using the geometric transform.

**Figure 3:**
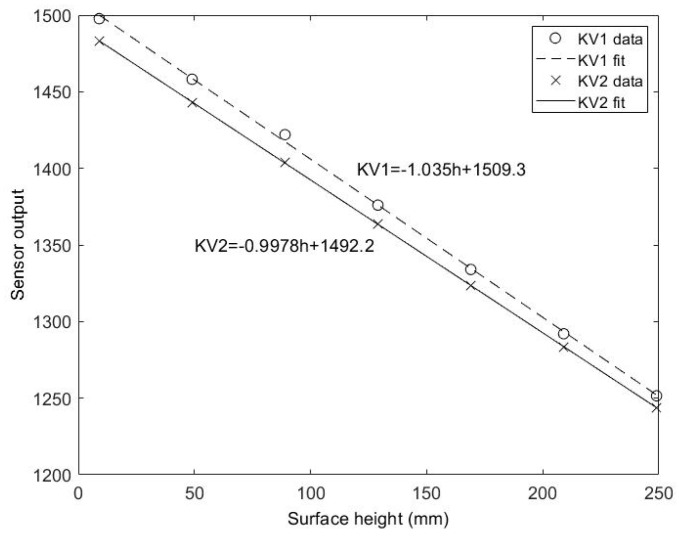
Depth calibration for KV1 and KV2.

The depth values recorded by the Kinect sensors nominally correspond to the distance from the sensor to the object being detected, in mm; however, since this was found to not be exact, the sensor output here was treated as a unitless value for which a calibration was required to assign it meaningful units in terms of mm. The depth data for the different vertical positions of the calibration board were therefore used to produce a linear depth calibration for each pixel location (example for the central pixel is given in Figure 3). All pixels showed a linear calibration with similar constants. Figure 3 also shows the calibration equation for the example pixel, showing that the sensor output relates approximately to the distance from the sensor to the surface. However, the offset shows that this distance is not from the front face of the sensor (9.3 mm offset for KV1, −7.8 mm offset for KV2), and more critically the gradient does not have a unity magnitude, so using the raw data would produce a 3.5% error in depth changes for KV1, and 0.22% for KV2. This further emphasises the superiority of the KV2 sensor for reliable scientific measurements, though a thorough calibration such as that in Figure 3 is still recommended. 

### 2.3. Accuracy of KV1 vs. KV2—Stationary Surface Measurement Accuracy and How to Improve It

**Figure 4:**
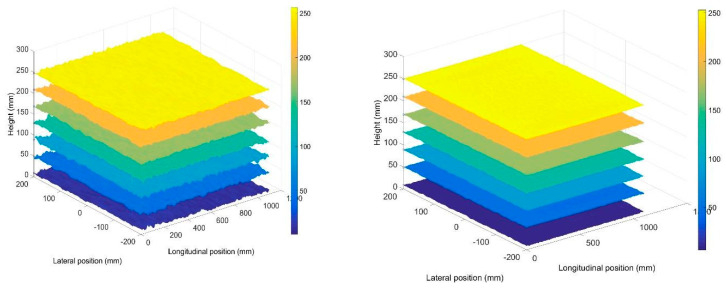
Measurements of solid surface by KV1 (**left**) and KV2 (**right**).

Solid surface data was used to assess the accuracy and variability in the depth measurement for both sensors. Figure 4 shows the time-averaged surfaces (from 30-s recordings) for seven different heights recorded by KV1 (left) and KV2 (right). The sensors were in the same configuration as in Section 2.2 with a vertical (downward) view direction. It can be seen that the spatial variability is significantly lower for KV2, and does not significantly vary with surface height (average spatial standard deviation across all time-averaged surfaces for KV1 = 1.88 mm, and for KV2 = 0.66 mm), suggesting the KV2 sensor to be around 3 times more reliable for time-resolved processes where surface “texture” is important.

**Figure 5:**
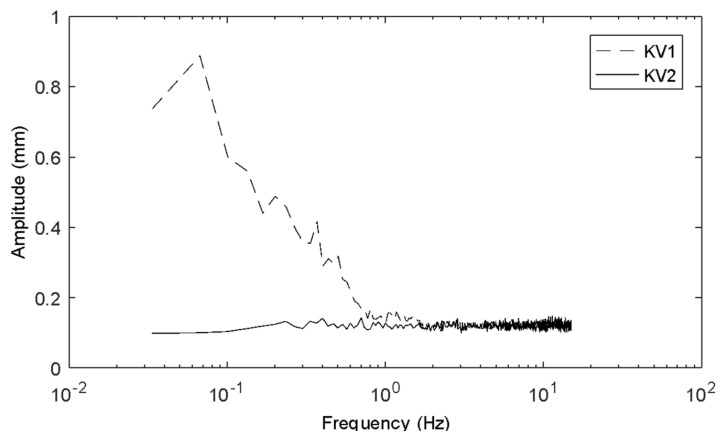
Frequency spectrum of noise of KV1 and KV2 for solid surfaces.

Figure 5 shows the frequency spectrum of the noise in the time series recorded by KV1 and KV2 for the stationary solid flat surface measurements. The spectrum was similar for all locations on the surface and all surface heights, so these spectra are spatially averaged. The ground truth in this case, since the surface was stationary, is zero amplitude across the full spectrum. It can be seen that KV2 exhibits a relatively flat spectrum, with no noise components above 0.2 mm in amplitude, whereas KV1 exhibits noise up to almost 1 mm, particularly below 1 Hz. This can be important for studies of low-frequency phenomena or single events such as dam breaks [54,55,56,57] where the behaviour of interest is in the order of mm. In these cases, it would be an order of magnitude more reliable to use a KV2 sensor.

**Figure 6:**
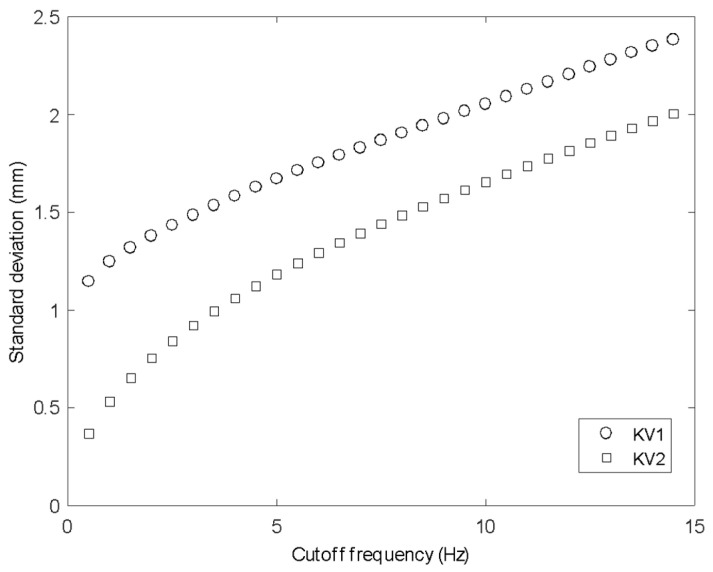
STD for reducing filter frequency cutoff for KV1 and KV2 for solid surfaces.

Figure 6 shows the standard deviation of a time series from one point on the surface as a function of the cutoff frequency of a third-order low-pass Butterworth filter applied to the data. This graph is similar for any point on the surface, so the figure shown is an average of the graph for each spatial location. It can be seen that with a cutoff frequency close to the Nyquist, the standard deviation tends toward the unfiltered values of 2.62 mm for KV1 and 2.04 mm for KV2. Depending on the free-surface dynamics of interest, the noise in the signal can thus be reduced by filtering to improve the signal-to-noise ratio. For example, a 1 Hz surface feature can be observed with system noise below 1.3 mm for KV1 and 0.6 mm for KV2. This further demonstrates that the KV2 sensor is preferable, particularly for measurement of small-scale surface features. 

**Figure 7:**
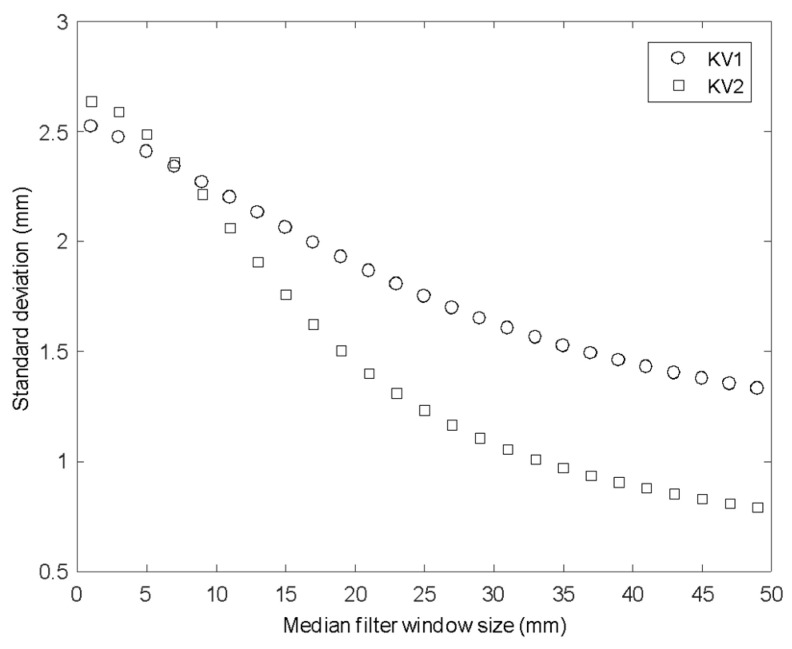
STD for increasing moving average window size for KV1 and KV2 on solid surfaces.

Another option for removing noise is to apply a spatial filter that is smaller than the smallest length scale of interest. Figure 7 shows the spatial standard deviation in surface height for a single instant in time for both KV1 and KV2, as a function of the window size of a two-dimensional median filter. It can be seen that the spatial noise for an instantaneous surface measurement is similar on the two sensors when unfiltered (2.52 mm for KV1 and 2.64 mm for KV2), but as the filtering window is increased, the KV2 data is significantly smoother, with standard deviation below 0.8 mm for a window size of 50 mm (where KV1 gives standard deviation of over 1.3 mm). 

### 2.4. TiO_2_ Concentration and Still-Water Stability Tests

To establish the optimal TiO_2_ concentration for the sensors to accurately capture the free surface, the calibration board was removed and the tank was replaced and filled with water to a depth of 140 mm. Nineteen different concentrations of TiO_2_ in approximately uniform increments were studied [41], ranging from 0% to 0.0162% by mass. The TiO_2_ used in this study was anatase titanium (IV) oxide from Acros Organics (Fair Lawn, New Jersey, United States) with molecular weight 79.88 kg/kmol. After TiO_2_ was added, the fluid was mixed by hand using a small paddle. Uniform mixing was visually observed to occur after 20 s but mixing was maintained for at least 1 min to ensure homogeneity of concentration. Any surface fluctuations were allowed to dissipate over a short time period and the Kinect data were then recorded for 10 s at 30 fps. 

**Figure 8:**
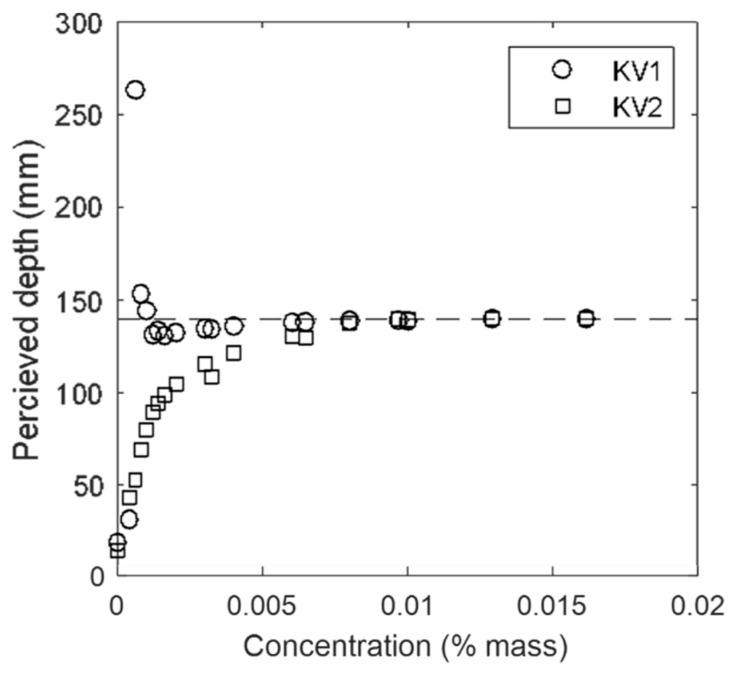
Perceived depth vs. concentration of TiO_2_.

Figure 8 shows the changes in time and space averaged depth detected by KV1 and KV2 as the concentration of TiO_2_ (%) was increased. While KV1 approached the correct value more quickly, neither sensor showed a reliable measurement until after a concentration of 0.01% by mass. Previous studies did not examine the accuracy of measurement as a function of TiO_2_ concentration [40,42,43], and did not state the TiO_2_ concentration used, so it is impossible to know whether those measurements were collected with an appropriate amount of TiO_2_. Martinez-Aranda et al. [41] stated a TiO_2_ concentration of 1.2%, which Figure 8 shows to be approximately 100 times greater than that which is necessary. 

**Figure 9:**
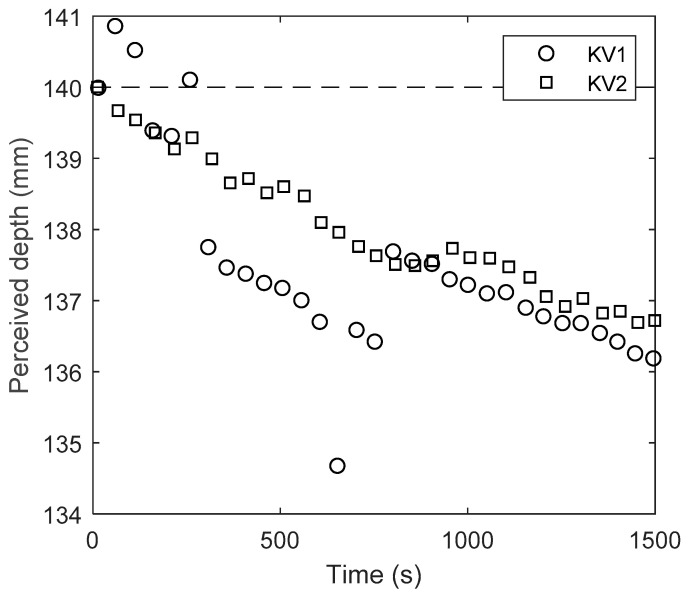
Perceived depth over time for TiO_2_ solution.

It is also important to understand the behaviour of TiO_2_ colourant within still water or low Reynolds number flows, where the TiO_2_ may settle under gravity. Przadka [50] found a TiO_2_ solution of 4% appeared to an optical camera system to have dropped by 0.23 mm after 30 min, but this may not be the same for the optimal 0.01% concentration, and also when sensed using infrared rather than visible light. Figure 9 shows the change in perceived depth over time for KV1 and KV2 after mixing the 0.01% of anatase TiO_2_ in the tank. Ten-second recordings were taken at intervals of approximately 1 min for a period of 25 min. The perceived depth can be observed to slowly decrease over time. The KV1 sensor shows more scattered perceived depths, which suggests that it may not be very reliable soon after the solution is allowed to rest. Previous studies have used KV1 [40,42,43], however, these results would suggest that KV2 is more reliable when there is a chance of TiO_2_ settlement. These data demonstrate two key findings: (1) KV2 again shows a more stable response than KV1; (2) there is a decrease in measured depth over time, meaning that fluid should be continuously mixed (perhaps by turbulence in the case of a turbulent flow) in order to maintain accuracy. The apparent settling is an order of magnitude faster than that measured by Przadka [50] using an optical camera rather than the infrared sensors employed by Kinect. This has implications for studies such as dam breaks, where the fluid may be resting behind the dam for an unspecified period of time before being released. 

## 3. Effect of TiO_2_ Concentration on Fluid Properties

Section 2 established a minimum TiO_2_ concentration of 0.01%. It is thought that overdosing can have a significant effect on the hydrodynamics, because a 1% concentration of TiO_2_ is reported to reduce the surface tension by almost 30% and increase the fluid viscosity by over 10% [46]. Viscosity proportionally affects the flow Reynolds number, which is crucial for understanding turbulence processes and also drag and energy losses resulting from flow around obstacles. Laiadi and Merzougui [58] showed that changes in surface tension can affect the free-surface profile in shallow flows, while Balabel and Alzaed [59] showed that changes in surface tension and viscosity can affect the propagation of the wave front in dam break scenarios. This may explain why Martinez-Aranda et al. [41] found that their experimental TiO_2_–Kinect data did not match the established model data, particularly in the vicinity of obstacles, where surface tension and viscosity effects would be more apparent. These experimental uncertainties are also apparent in comparison with other models [60]. 

Przadka et al. [50] found anatase TiO_2_ to marginally affect wave properties, but this was for a transient wave of larger magnitude than the waves often of interest in turbulent flows. Hence, this section will systematically explore the effect of anatase TiO_2_ concentration on surface tension and gravity wave behaviour for small-scale, continuously generated waves. The relevance of this is that the effect of TiO_2_ may then be inferred for a given concentration. It can therefore be used to elucidate the potential impact on previous studies that used TiO_2_ indiscriminately, and to inform experimental design of future TiO_2_–Kinect measurements.

### 3.1. Effect of TiO_2_ on Surface Tension

**Figure 10:**
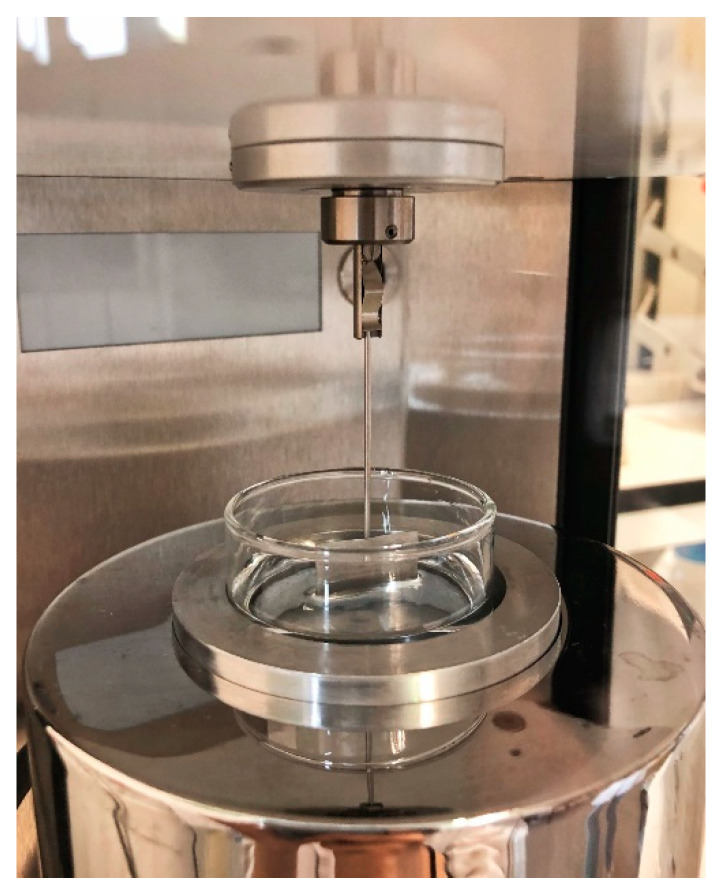
Surface tension measurement by KRUSS tensiometer with plate method.

The liquid surface tension was measured using a KRUSS tensiometer (model no. K11MK4) (Figure 10) with the plate method. Samples of water with different concentrations of TiO_2_ (0–2%) were prepared and well mixed before the measurement. A plate was lifted up from the surface of the sample in the container and the force required to raise the plate from the liquid surface was measured to determine the surface tension. Each measurement was repeated five times and averaged.

**Figure 11:**
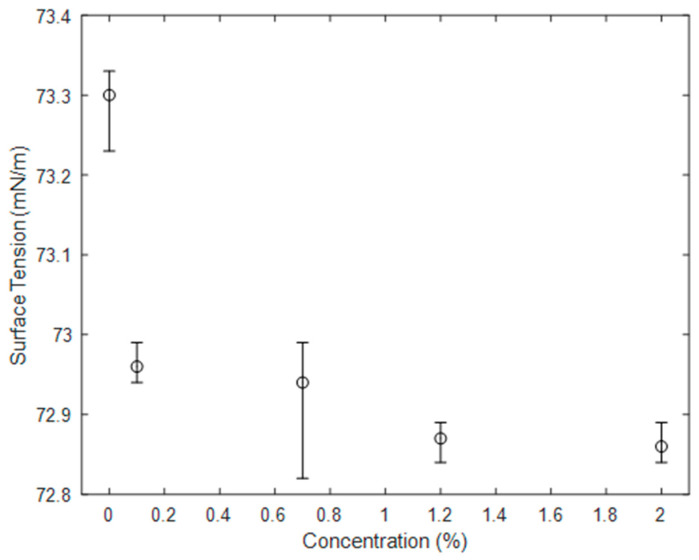
Surface tension of water as a function of TiO_2_ concentration. Error bars represent maximum and minimum of 5 repeats, markers represent the average.

Figure 11 shows the surface tension as a function of TiO_2_ concentration. It can be seen that even small concentrations cause a change in surface tension, with concentrations above 1% reducing the surface tension by over 0.5%. This difference may be enough to substantially affect fluid behaviour in the capillary wave regime or where a fluid is in contact with a solid obstacle. 

### 3.2. Effect of TiO_2_ on Gravity–Capillary Waves

**Figure 12:**
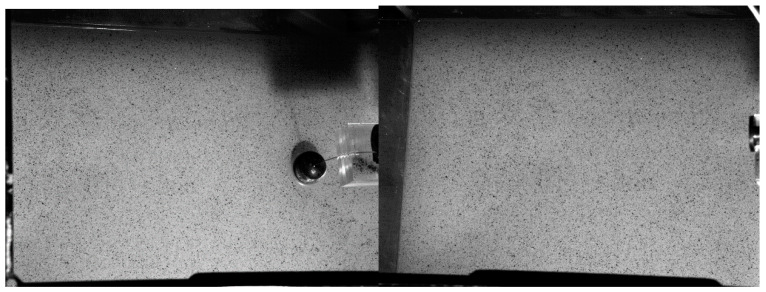
Two views of two DIC cameras from two directions.

The purpose of this test was to investigate the effect of anatase TiO_2_ concentration on the behaviour of gravity–capillary waves on a still-water surface. Due to the stability issues with TiO_2_–Kinect measurement in still water and low TiO_2_ concentrations, the water surface was characterised using a Digital Image Correlation system (DIC) Q-400 (www.dantec-dynamics.com), which only required the background to be broadly white, with some darker floating tracers at the free surface. DIC is an optical measurement method based on stochastic pattern (speckles) recognition on the object to be measured. It is widely used in full-field displacement and strain measurement. The DIC system observes speckles with two cameras from different directions, and the 3D measurement can then be achieved by identifying and tracking these speckles. An amount of 5000 mL of tap water with different concentrations of TiO_2_ (0.01–1.2% by mass) was added to the tank with horizontal dimensions 355 mm × 210 mm. Black pepper was randomly distributed on the surface of the liquid to be used as speckles floating on the fluid surface (Figure 12). A continuous wave was excited by a 25 mm diameter sphere moving up and down sinusoidally, connected to a servo motor controlled by an Arduino Uno microprocessor (Arduino AG, Italy) at a frequency of 2.5 Hz and amplitude of 0.25 mm. Tests were repeated 10 times for each concentration and a 10 s period was recorded for every measurement.

**Figure 13:**
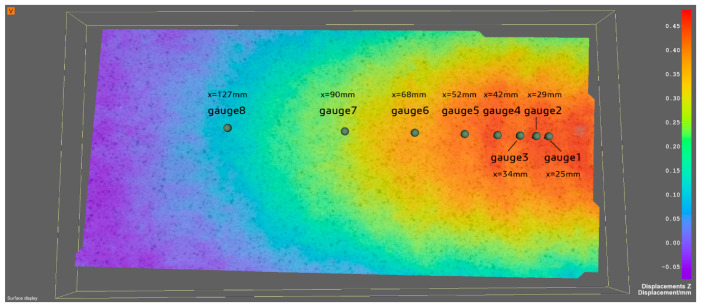
A section of evaluated displacement in z-direction by ISTRA 4D (x is the distance between the gauge point and centre of the sphere).

The displacement of the wave in the vertical direction was evaluated from the videos of the two cameras. Eight gauge points were chosen along the direction of the travelling wave, with different distances from the centre of the sphere generating the waves as illustrated in Figure 13. 

**Figure 14:**
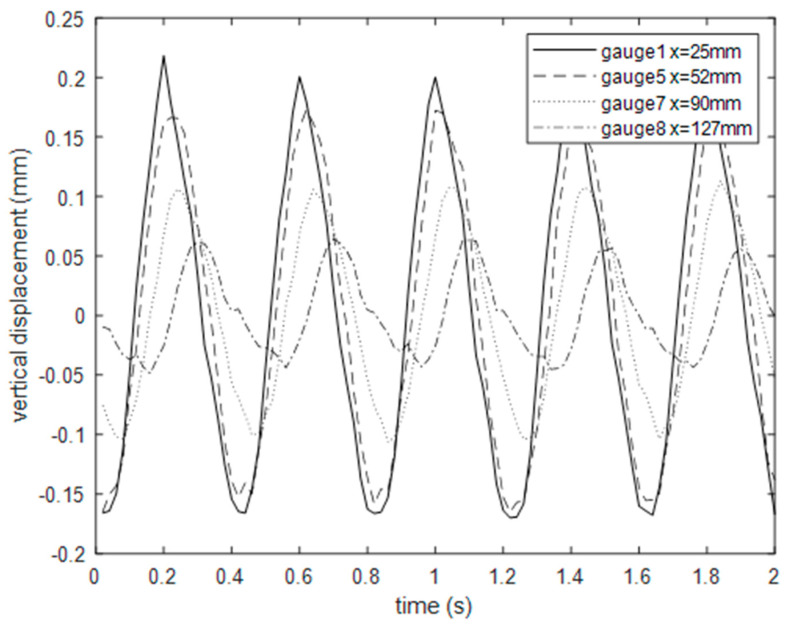
Time series of vertical displacement of four gauge points for concentration 1.2%.

The vertical displacement of the eight chosen gauge points was computed by the Dantec dynamics software Istra-4D version 4.4.7.507 (the control software of the system Q-400). The exported data from ISTRA 4D in HDF5 format were imported into MATLAB R2019a and then processed. The wave height decreases as the gauge point moves further away from the sphere (centre of the wave), as shown in Figure 14. A phase shift is also recognisable, illustrating the translation and celerity of the wave. 

**Figure 15:**
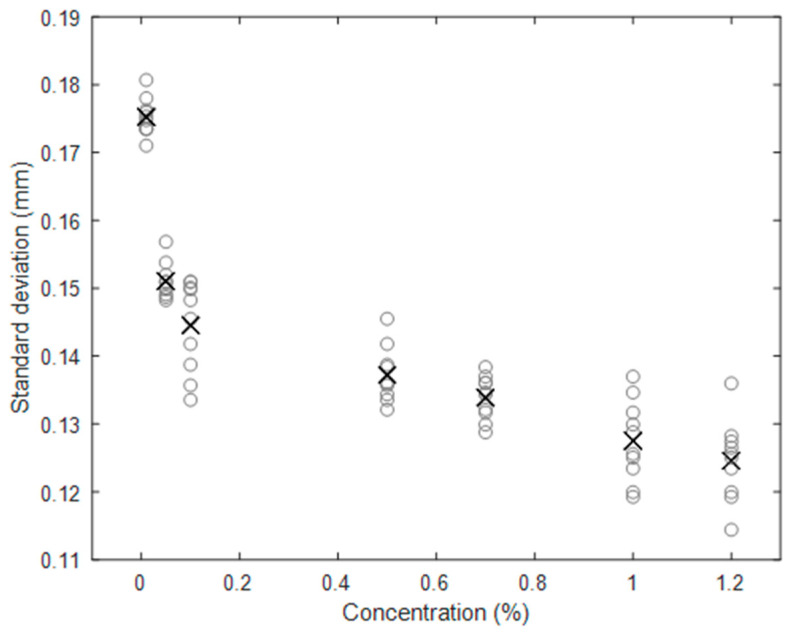
Standard deviation of wave fluctuation at gauge point 1 for different concentrations. Circles represent 10 repeats for each concentration, crosses represent the mean.

**Figure 16:**
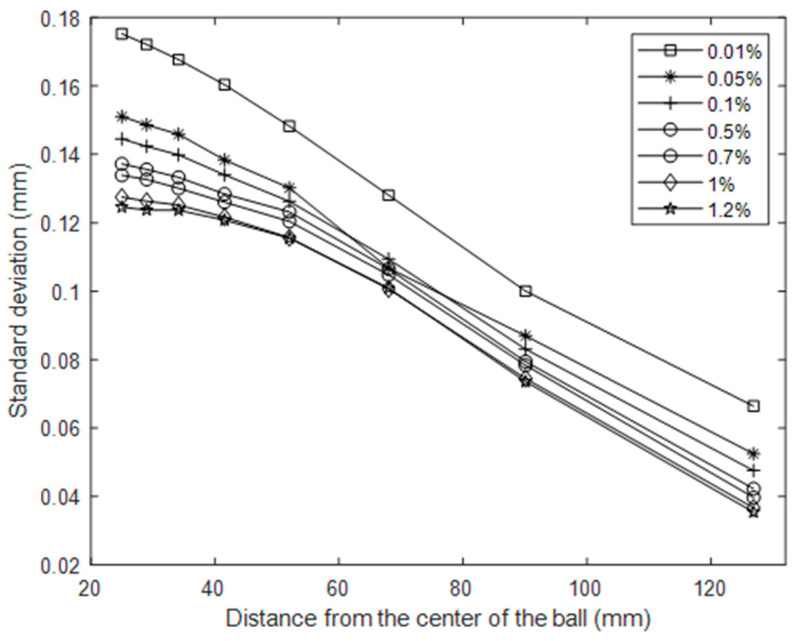
Standard deviation of wave fluctuations over distance for a range of concentrations.

Figure 15 shows the standard deviation of the recorded wave signal in mm for gauge point 1, for 10 repeated measurements at each concentration. The cross markers show the mean value for each concentration. It is apparent that the wave height is substantially affected by the TiO_2_ concentration. For a TiO_2_ concentration of 1%, the wave height is reduced by more than 25% compared with a 0.01% concentration. This has significant impact for all studies utilising TiO_2_–Kinect measurements to characterise free-surface dynamics. Figure 16 shows the mean value of standard deviation for each concentration as a function of distance from the wave centre. It is clear that at all distances, the impact of TiO_2_ concentration on wave height is clearly apparent.

**Figure 17:**
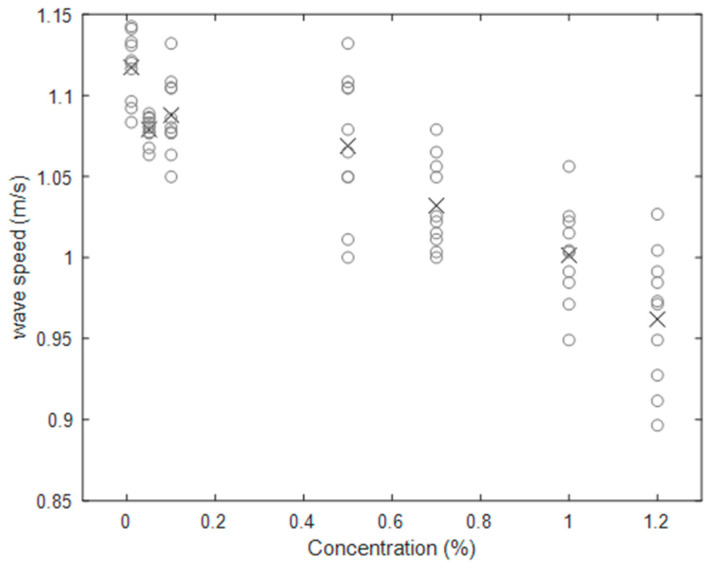
Averaged wave speed from 10 repeats versus different concentrations. Circles represent 10 repeats for each concentration, crosses represent the mean.

Figure 17 shows the phase velocity of the wave, measured between gauge points 1 and 8, for each concentration. The wave speed was calculated by the ratio of separation of gauges (1 and 8) and the time phase lag. The phase shift was determined from analytical signal theory (Hilbert transform).

There is a clear trend in TiO_2_ concentration reducing the phase speed of the wave, with a 1% concentration reducing the phase speed by as much as 13.91% compared to 0.01% concentration. This again indicates that the behaviour of water surfaces with high TiO_2_ concentrations (>0.01%) may be substantially different to that of water alone, meaning studies of water waves that use higher concentrations cannot be directly interpreted or applied unless the effect of the high concentration is carefully assessed. 

## 4. Conclusions and Recommendations

This study examined the use of titanium dioxide with two generations of the Microsoft Kinect sensor, KV1 and KV2, in order to evaluate their performance against an ideal flat surface. Studies were conducted to establish the optimal anatase TiO_2_ concentration and sensor choice, and presented evidence that the fluid properties can be substantially affected by the TiO_2_. Results obtained can be summarised as follows:

- KV2 is more accurate and more reliable spatially and temporally for scientific applications.
- A TiO_2_ concentration of at least 0.01% is required for reliable Kinect measurements of surface shape.
- TiO_2_ concentration above 0.01% substantially affects fluid properties and must be taken into account if using TiO_2_-Kinect-derived data for model validation or other practical purposes.
- TiO_2_ of >1% is more significantly affected, showing a 27.85% reduction in gravity wave height and a 13.91% reduction in phase speed compared with a 0.01% concentration. It is strongly recommended to use the lower concentration to more closely represent pure water dynamics.
- TiO_2_ must remain well mixed, so this technique is not recommended for low Re flows or transient processes involving still water.

These results confirm that it is essential to consider the effects of TiO_2_ concentration before TiO_2_-Kinect data can be reliably interpreted, and suggest the employment of KV2 sensors for future studies with a TiO_2_ concentration of 0.01%.

If the above limitations and considerations are properly accounted for, this data does support the use of the TiO_2_-Kinect technique, under carefully controlled and understood conditions, to measure dynamic free-surface roughness. Future research should also include a frequency sensitivity test to characterise the response to stimuli at different frequencies, perhaps by measuring gravity waves in water coloured with TiO_2_, or fluctuation of a solid surface. The TiO_2_-Kinect method may play a pivotal role in the development of a suite of fast, accurate and cost-effective free-surface measurement techniques [61,62,63] that could enhance the understanding of underlying phenomena in rivers and oceans as well as flooded urban areas as climate change increases flood risk.

## Figures and Tables

**Table 1:** Studies employing the Kinect–TiO_2_ method.

Paper	Author	Kinect Version	Colourant
Free-surface flows from Kinect: feasibility and limits. Proc., Forum on Recent Developments in Volume Reconstruction Techniques Applied to 3D Fluid and Solid Mechanics (FVR 2011), Chasseneuil, France 2011. [42]	Combès, B., Guibert, A., Memin, E., Heitz D.	1	“white dye”, concentration not stated
Remote sensing of environmental processes via low-cost 3D free-surface mapping, *4th IAHR Europe Congress*, Liege, Belgium, 27–29 July 2016. [39]	Nichols, A., Rubinato, M.	1	TiO_2_, concentration not stated
P. Towards transient experimental water surfaces: A new benchmark dataset for 2D shallow water solvers. *Advances in Water Resources*, 121, 130–149, 2018. [40]	Martinez-Aranda, S., Fernandez-Pato, J., Caviedes-Voullieme, D., Garcia-Palacin, I., Garcia-Navarro, P.	1	TiO_2_, concentration 1.2%
Measuring surface gravity waves using a Kinect sensor. *Journal of Mechanics – B/Fluids*, 2018. [43]	Toselli, F., De Lillo, Onorato, M., Boffetta, G.	1	“commercial paint”, concentration 1%
Towards transient experimental water surfaces: strengthening two-dimensional SW model validation. *13th International Conference on Hydroinformatics*, Palermo, 1–6 July 2018. [41]	Martinez-Aranda, S., Fernandez-Pato, J., Caviedes-Voullieme, D., Garcia-Palacin, I., Garcia-Navarro, P.	1	TiO_2_, concentration 1.2%
